# Extinction of outcome-specific Pavlovian-to-instrumental transfer (PIT), instrumental outcome devaluation, and reward-related attentional capture are predicted by affect-driven impulsivity

**DOI:** 10.3758/s13420-025-00676-1

**Published:** 2025-05-14

**Authors:** Felisa González, Francisco Garre-Frutos, Irene Hinojosa-Aguayo, Geoffrey Hall

**Affiliations:** 1 Mind, Brain, and Behavior Research Center (CIMCYC), University of Granada, Granada, Spain; 2 Department of Experimental Psychology, University of Granada, Granada, Spain; 3 Department of Psychology, University of York, York, UK; 4 School of Psychology, University of New South Wales, Sydney, NSW Australia; 5 Present Address: Google User Experience Research, Brandschenkestrasse 100, 8002 Zurich, Switzerland; 6 Department of Psychology, University of Granada, Campus of Cartuja, 18071 Granada, Spain

**Keywords:** Attentional sign-tracking, Extinction, Negative urgency, Pavlovian instrumental transfer, Emotion regulation, Incentive salience

## Abstract

In two online experiments, we aimed to study the relationship between emotion dysregulation and persistence of incentive salience attributed to reward cues. Participants’ negative urgency (NU) was assessed before they completed a value-modulated attentional capture (VMAC) task measuring incentive salience as attentional sign-tracking. This consisted of two phases – rewarded and unrewarded – to evaluate the persistence of the VMAC effect. Subsequently, a Pavlovian-to-instrumental transfer (PIT) procedure was employed as another measure of incentive salience. In Experiment 1 both outcome-specific and general PIT effects were assessed, along with the impact of instrumental outcome devaluation (OD). Experiment 2 focused on the effect of Pavlovian extinction on specific PIT. Both outcome devaluation and extinction are indices of implicit emotion regulation. In Experiment 1, the OD index showed a significant positive correlation with specific PIT and a negative correlation with the NU score. In Experiment 2, the extinction index of specific PIT, linked to the level of explicit knowledge of the contingencies, correlated negatively with NU. The VMAC effect and its persistence showed correlations with NU, positive and negative, respectively (Experiment 1). No relationships were found between any measure of VMAC and OD or PIT effects in any of the experiments. These findings suggest that difficulties in emotion regulation are associated with increased attentional sign-tracking and can hinder action control and selection. These phenomena may be governed by distinct mechanisms, with the VMAC effect being more automatic and the specific PIT effect exhibiting varying degrees of goal-directed behavior depending on the effectiveness of implicit emotion regulation strategies.

**Supplementary Information:**

The online version contains supplementary material available at 10.3758/s13420-025-00676-1.

## Introduction

Adaptive, flexible reward-seeking behavior depends on the integration of both cue-outcome (Pavlovian conditioning) and action-outcome (instrumental conditioning) knowledge, which interact in the Pavlovian-to-instrumental transfer (PIT) procedure (Estes, 1943). Emotion dysregulation, defined as a failure in the operation of processes, conscious or unconscious, aimed at modifying the trajectory of an emotion, can result in emotional responses that are excessive, inappropriate, or insufficient (Etkin et al., 2015), potentially affecting both learning processes. Under certain conditions or in vulnerable individuals, emotion dysregulation may be linked to excessive *incentive salience* (“wanting”) attributed to reward cues, triggering more intense motivation to pursue reward (Peciña & Berridge, 2013). In the present work we aim to investigate the relationship between emotion dysregulation and excessive incentive salience attributed to reward cues in both Pavlovian and instrumental contexts.

### Incentive salience: Attentional sign-tracking in Pavlovian scenarios

Excessive incentive salience can make the cues attract attention by themselves, becoming desirable, triggering approach as well as reward-seeking responses, and thus acting as “motivational magnets” (Berridge, 2001; Berridge & Robinson, 2003; Olney et al., 2018), even at the expense of missing or neglecting current goals (but see Derman et al., 2018, for an expectancy-mediated account of sign-tracking behavior as a measure of incentive salience in rodents sensitive to outcome devaluation).

Individual differences in the tendency to attribute incentive salience to reward cues have been linked to the *sign-tracking* (ST) phenomenon. When discrete and localizable stimuli are utilized in appetitive Pavlovian conditioning, ST (Hearst & Jenkins, 1974) is described according to the conditioned response exhibited by rats toward reward cues (Derman et al., 2018). For instance, a stimulus (such as the insertion of a lever) precedes the delivery of food in the magazine, becoming a predictor of food availability and triggering a conditioned response (magazine entry) directed to the place where the food (the goal) will be found. For sign-trackers, however, the cue additionally acquires *incentive value*, directing behaviors to the conditioned stimulus (CS) itself (the lever) as if it were a food surrogate, such as sniffing and nibbling (Anselme et al., 2013). While ST could be beneficial in certain scenarios by, for example, increasing attention to cues signaling important outcomes such as food or water, it may be counterproductive when it shows inflexibility, resistance to extinction, or insensitivity to other changes in CS-unconditioned stimulus (US) contingency (such as reversal learning) or the value of an outcome (outcome revaluation). As reviewed by Colaizzi et al. (2020), ST-conditioned responses, in contrast to goal-tracking, show greater resistance to extinction, increased susceptibility to reinstatement, and persist even when they result in an adverse outcome or loss of reward. Although ST is well established in animal models as a plausible index of excessive incentive salience (Anselme et al., 2013), its direct application to individual differences in human subjects remains unclear.

Evidence suggests that ST behavior may be estimated in humans. For instance, Garofalo and di Pellegrino (2015) measured eye gaze during a Pavlovian conditioning task (monetary reward) to categorize participants as either sign-trackers or, alternatively, goal-trackers. Similarly, attentional ST has been studied through the value-modulated attentional capture (VMAC) effect (Anderson et al., 2011; Le Pelley et al., 2015). VMAC is typically measured using the additional singleton task (Theeuwes, 1992), although there are several variants (see Colaizzi et al., 2020). In the experiments reported here, we focus on a paradigm similar to the tasks employed in animal models: the reward cues are both task-irrelevant and response-independent. Consequently, attentional capture in this context is thought to be driven by Pavlovian rather than instrumental conditioning (Le Pelley et al., 2015).

In this version of the task (please refer to the *Procedure* section for further details), participants are presented with a uniquely shaped target among other non-targets of a different shape, forming a circular search array. To earn points, participants must determine the orientation (vertical or horizontal) of a line within the target while ignoring non-targets in the same stimulus array (e.g., Garre-Frutos et al. 2024; Watson et al., 2019). The optimal strategy is to ignore the distractors since attending to them would slow down the response, earning fewer points. However, participants tend to focus on the distractors, especially when they signal an increase in reward magnitude (condition high). On average, correct responses are typically slower than those of the control conditions without a corresponding trade-off in accuracy. This pattern suggests that attentional capture is modulated by the incentive value of the “high” cue, and for this reason, VMAC^1^ is considered appropriate for studying incentive salience as attentional sign-tracking in a Pavlovian scenario.

### Incentive salience in action control and selection: Pavlovian-to-instrumental transfer (PIT)

The examination of reward-seeking behavior in instrumental scenarios often involves using the PIT procedure (see Cartoni et al., 2016, Holmes et al., 2010, for reviews of animal studies; Mahlberg et al., 2021, for human studies; and Garbusow et al., 2022, for studies with a focus on human psychopathology), which may be considered a relatively pure assay of incentive salience (Peciña & Berridge, 2013). When it comes to investigating human learning, especially in the context of subclinical or clinical mental disorders, the use of the PIT procedure is a relatively recent field of research (Garbusow et al., 2022).

While there are several variants of the PIT task, the basic paradigm typically consists of three phases. In the initial two phases, participants undergo training in instrumental (R-O) and Pavlovian (S-O) conditioning (in any order), followed by a transfer test in the third phase. Following each of the two training phases, participants’ explicit knowledge about the relevant associations may be assessed. Finally, on the transfer test, participants freely perform the instrumental responses while the Pavlovian cues are occasionally presented at different times during the session. This phase is typically conducted in nominal extinction to prevent further learning; that is, participants are told that they are still earning outcomes, but these are not displayed on the screen (for a more detailed description, please refer to the *Procedure* section).

This paradigm (e.g., Hinojosa-Aguayo & González, 2020; Morris et al., 2015) may allow for estimating both outcome-specific and general PIT effects (see Table 1). Specific PIT is observed as a bias in choice behavior, where the presence of a Pavlovian cue selectively increases the rate of the particular response with which it shares the outcome. On the other hand, general PIT is evident when a stimulus previously paired with a third outcome, not presented during instrumental training, produces a non-selective invigorating effect on general responding compared to baseline.

**Table 1:** Experiment 1: Experimental design of the Pavlovian to instrumental transfer (PIT) effect and instrumental outcome devaluation test (following Hinojosa-Aguayo & González, 2020 after Morris et al., 2015)

Instrumental training	Pavlovian training	Transfer test	Outcome devaluation	Devaluation Test
R1 - O1	S1 - O1	S1: R1 (Same) vs. R2 (Diff)		
R2 - O2	S2 - O2	S2: R1 (Diff) vs. R2 (Same)	Devaluation (O1 or O2)	R1 vs. R2
	S3 - O3	S3: R1, R2 (CS+)		
	S4 – no outcome	S4: R1, R2 (CS-)		

### Emotion dysregulation and the persistence of attentional sign-tracking and reward-seeking behavior

As previously mentioned, emotion dysregulation may lead to inappropriate emotional responses (Etkin et al., 2015). In this context, the persistence of attentional sign-tracking and reward-seeking behavior, even when the S-O contingency or the incentive value of the outcome is reduced, may serve as a proxy for maladaptive cue reactivity, suggesting an inability to integrate and update new information. It is important to note that both extinction and outcome devaluation have been recognized as forms of implicit emotion regulation (Braunstein et al., 2017) – strategies aimed at modifying one’s emotional state, primarily driven by incidental model-free processes, which can be assessed through objective computerized behavioral tasks (Muela et al., 2023). In addition, emotion dysregulation as a trait can be measured using questionnaires. For instance, *Negative urgency*, a factor of the UPPS-P scale (see *Methods* section), captures emotional impulsiveness, favoring immediate solutions that provide short-term emotional relief. This reflects an inefficient strategy for long-term emotion regulation (King et al., 2018) and is therefore considered a form of generalized emotion dysregulation (Jara-Rizzo et al., 2019).

### The present work

Given the above, we aim to examine the relationships between (1) failures in implicit emotion regulation processes involving behavioral persistence, such as instrumental outcome devaluation (Experiment 1) and Pavlovian extinction (Experiment 2); (2) negative urgency, as an explicit measure of generalized emotion dysregulation; and (3) indices of incentive salience, measured through attentional sign-tracking (VMAC) and specific PIT. In both experiments, we also evaluated the persistence of the VMAC effect when the reward was omitted and its relationship with negative urgency.

Additionally, in Experiment 1, we aimed to replicate previous findings regarding the relationships between instrumental outcome devaluation, specific PIT, and negative urgency (Hinojosa-Aguayo & González, 2020, Exp. 1). In Experiment 2, a conceptual replication of Experiment 2 in Seabrooke et al., 2018 using our task, we sought to extend these findings by examining the effect of Pavlovian extinction on specific PIT and exploring any potential relationships with negative urgency.

Given previous results (Hinojosa-Aguayo & González, 2020, Exp. 1), in Experiment 1 we expected to find a positive relationship between the indices of specific PIT and instrumental outcome devaluation,^2^ as well as negative correlations between both of these and the negative urgency score. Regarding the VMAC task the predictions were less clear. We broadly followed the procedure used by Watson et al. (2019), with an acquisition phase followed by another in which reward (points) was omitted on all trials (akin to an extinction procedure), with only feedback on accuracy provided to participants. Previous studies, using a reward-only variant of the VMAC task and the total score on the short UPPS-P impulsivity scale, have found a positive correlation between the VMAC effect and impulsivity in participants with addiction-related and compulsive-obsessive behavior (Albertella et al., 2019a). This is consistent with the idea that the VAMC effect may be related to problems of behavior control, but it needs to be tested in a healthy sample. We expected to find a VMAC effect during the acquisition phase that was positively related to negative urgency score. However, we were unable to make a clear prediction regarding the effect of reward-omission. If observed, as an index of flexibility, we would expect to find significant positive relationships with indices of instrumental outcome devaluation and specific PIT, along with a significant negative relationship with the negative urgency score. For clarity, predictions regarding Experiment 2 will be presented later, following the discussion of Experiment 1. In both experiments, participants first completed the VMAC task. Twenty-four hours later, they were sent another link to the PIT task (see Procedure for details).

## Experiment 1

### Methods

#### Participants

Participants were recruited through advertisements made available to students and members of the wider community of the University of Granada. Due to the potential introduction of noise in the data in an online study, we increased the target sample size from 48 (sufficient for yielding differential sensitivity in a previous study performed in our laboratory; see Hinojosa-Aguayo & González, 2020, Exp. 1) to 60 participants. No specific a priori power analysis was conducted. The analyses only included participants who completed the sociodemographic questions, the UPPS-P, and the two experimental tasks (VMAC and PIT), and declared to be fluent in Spanish, have normal or corrected-to-normal vision, and normal color perception. Additionally, only participants whose VMAC data met the inclusion criteria were considered in the reported analyses. Of the 75 participants who chose to take part in the study, 14 did not complete the two tasks. From the remaining 61 participants, we filtered out those with accuracy lower than 70% in the VMAC task. The final sample consisted of 58 participants (15 males), 88% of whom were university students. Their average age was 22.0 years (*SD* = 2.8, range 18–29 years). At the end of the study, participants received compensation of between 3 and 8 euros based on their performance on the VMAC task.

#### Materials

##### Spanish adaptation of the short version of the UPPS-P questionnaire

(Cándido et al., 2012). This questionnaire contains 20 items, four for each factor considered in the five-factor model of impulsiveness (Whiteside & Lynam, 2001): negative and positive urgency, (lack of) premeditation, (lack of) perseveration, and sensation-seeking. We focused on negative and positive urgency factors which are of special interest as they are considered to be affect-driven impulsiveness factors, particularly negative urgency, which has been identified as a common transdiagnostic factor for addictive and other externalizing disorders characterized by emotion dysregulation (Quintero et al., 2020; see also Muela et al., 2023). Reliability estimations in our sample for negative and positive urgency subscales (Cronbach’s alpha) in the present experiment were.91 and.84, respectively.

##### Value-modulated attentional capture (VMAC) task

The task was programmed in OpenSesame software (Mathôt et al., 2012) and hosted in JATOS (Lange et al., 2015). As the task was completed online, each participant estimated their distance from the screen using the virtual chinrest procedure devised by Li et al. (2020). In this procedure, the dimensions of the stimuli are adjusted based on the eccentricity of items in the search display. The experimental task was adapted from that used by Watson et al. (2019), with the exception that the reward-omission phase comprised 12 instead of two blocks, matching the acquisition phase (see also Garre-Frutos et al., 2024). The first 24 trials served as practice trials similar to those used in the acquisition phase, except that the colors used for Low and High trials were different to those used in the main experimental task. Participants were instructed to respond as accurately and quickly as possible, as available points depended on their performance level. Additionally, they were informed that a bonus trial would occur when one of the circles appeared in a particular color (associated with the High condition). See below for further details. Instructions provided to participants for this task are available in the Appendix.

Following the practice trials, the acquisition phase began. A central fixation cross appeared on the screen before the search display was shown. Six shapes (2.3º × 2.3º visual angle) were evenly arranged to form an imaginary circle (10.1º). Five of these shapes were circles containing lines tilted 45º randomly to the right or left. The target was a gray diamond containing a line oriented randomly horizontally or vertically. On singleton Absent trials, all shapes were gray. On Low singleton trials, one of the circles was randomly colored (either blue or orange for some participants or green or pink for others, with random assignment), while the alternative color signaled the High singleton trials. The locations of the target and the distractors were random on each trial. Participants were tasked with determining the orientation of the line within the diamond (horizontal or vertical) as quickly and accurately as possible by pressing the “V” or the “H” keys, respectively. Therefore, the color of the distractors played no instrumental role in the task. Each block comprised 24 trials: four without singleton distractors (condition Absent), ten corresponding to the Low condition, and ten to the High condition. Participants earned 0.1 points for every millisecond that their response time (RT) was below 1,000 ms on the Absent and Low trials. On the High trials, the points were multiplied by 10. Responses with an RT longer than 1,000 ms were not rewarded, and errors led to a loss of points equivalent to what would otherwise have been earned. The search display remained until the participant responded, or for 2,000 ms, followed by feedback for 700 ms, indicating the number of points earned or lost for correct or incorrect responses, respectively. The intertrial interval was 1,200 ms. After the acquisition phase, the unrewarded phase began. Unlike Watson et al. (2019), the length of this phase was equal to the acquisition phase, with 12 blocks of 24 trials each. Otherwise, the task remained the same – to determine the orientation of the line within the diamond. However, no points were awarded based on performance, and participants were informed of this aspect of the procedure. Only feedback on accuracy was provided.

#### Pavlovian to instrumental transfer (PIT) task

##### Initial level of hunger and outcome pleasantness rating

Before starting the PIT task, hunger and pleasantness were assessed using a 7-point Likert-type response scale ranging from 0 (not at all) or 7 (extremely).

*PIT* *task*. For the present study (see Table 1), we slightly modified the computerized task used in Experiment 1 of Hinojosa-Aguayo and González (2020), which was an adaptation of those employed by Morris et al. (2015) and Quail et al. (2017). The task was programmed using the online experimental platform “Labvanced” (https://www.labvanced.com). Instructions provided to participants for this task are available in the Appendix.

##### Instrumental training

Participants were instructed to collect as many free food snacks as they could from a virtual vending machine. To do so, they were required to tilt the machine to the right and to the left using the “up” and “down” arrow keys of the keyboard (R1 or R2, randomized across participants) using the index finger of their dominant hand. The outcomes (Os) were images of three snacks (M&Ms chocolates, crisps, and a popular chocolate cookie in Spain) that were randomly assigned to O1, O2, and O3. During the instrumental phase, participants performed R1 and R2 to obtain O1 and O2. They completed six blocks of trials on which they freely performed the two responses, with reinforcement following a random ratio schedule (the number of consecutive responses required to obtain the outcome varied randomly between 5 and 10). The image of the outcome appeared on the screen for 1 s. Once three outcomes were obtained, participants were asked to identify which key they should press to obtain a particular outcome whose image was displayed on the screen. They were given feedback, “Correct” or “Incorrect,” which remained for 1 s. This procedure, inquiring about the R-O assignment, was repeated six times. The instrumental phase concluded when the participants correctly answered the six questions consecutively for a given block.

##### Pavlovian training

During this phase, participants observed passively as the vending machine was intermittently lit with one of four colors: blue, green, red, and yellow. They were informed that each color signaled whether the machine was currently empty or filled with one of three snacks that could fall freely without the need for tilting the machine. Two colors (S1 and S2) signaled that the instrumental outcomes (O1 and O2, respectively) could freely fall from the machine; a third color (S3) signaled the free availability of a third outcome (O3) that was not present during the instrumental phase but shared motivational value with the stimuli used during this phase; and finally, a fourth color (S4) signaled that the machine was empty. The assignment of the four colors to S1, S2, S3, and S4 was randomized for each participant. There were four blocks of four trials each. On each trial, an uncolored image of the vending machine was displayed on the screen. Subsequently, the machine was lit with one of the four colors for 3 s, and the image of an outcome appeared during the last 2 s of this period before returning to the uncolored image. The intertrial interval varied between 1 s and 3 s. The order of the four colors in each block was randomized. After each block of trials, the image of the vending machine illuminated with one of the four colors appeared, and participants were given a multiple-choice question. They were asked to identify which of the four possible outcomes corresponded to that color, in random order, after which feedback was given for 1 s. After completing the four acquisition blocks, they were presented with a further set of similar questions asking about the four S-O associations without feedback. A score of explicit S-O knowledge was calculated based on the percentage of correct responses.

##### PIT

Participants had to tilt the vending machine to obtain the snacks, freely performing R1 and R2, but were advised that the outcomes would not appear on the screen this time (nominal extinction). Participants completed six blocks of four trials, with an ITI varying between 8 and 16 s. The vending machine appeared in the original, uncolored version during this interval, with the final 6 s constituting the preCS period. During each trial, the vending machine was lit with one of the four colors (in random order) for 6 s (the CS period) before returning to the uncolored version. To assess outcome-specific PIT, R1 was labeled as the *Same* response when performed during the S1 CS-period, and as *Different* when performed during the S2 CS-period. Conversely, R2 was labeled using the opposite assignment. To evaluate general PIT, R1 and R2 were collectively treated as “responses” and their total numbers were summed when performed during the S3 and S4 CSs-periods, and when computing the average number of responses during the preCS period.

##### Outcome devaluation

One of the two instrumental outcomes (O1 or O2) was randomly chosen and devalued by presenting a gif image in which two cockroaches run over the snack for approximately 10 s. Subsequently, participants completed the outcome devaluation test (lasting 120 s) in which they freely performed R1 and R2 in extinction. The participants then re-evaluated the pleasantness levels of the three outcomes, as well as their level of hunger.

#### Procedure

The protocols of the experiments reported here were approved by the Ethics Committee on Human Research of the University of Granada (approval number 3022/CEIH/2022). Both studies were advertised to students and members of the wider community of the University. Those who agreed to participate first completed a survey programmed in Limesurvey (https://www.limesurvey.org), which included the informed consent form, sociodemographic questions, the questionnaires,^3^ and the link to the VMAC task. Once participants completed this part of the study, they were sent another link to the PIT task 24 h later and were given 48 h to complete this task. This time delay was implemented to prevent participants from performing the two tasks consecutively and to mitigate potential carry-over effects such as tiredness or disengagement from the second task, which might negatively affect performance. After finishing the PIT task, participants were finally contacted to action the payment of the incentive according to their performance on the VMAC task.

### Results and discussion

#### Statistical analyses

Repeated-measures (RM) analyses of variance (ANOVAs) were conducted to determine main effects and interactions. Greenhouse-Geisser correction was applied when the sphericity assumption was violated, and effect sizes were estimated using η^2^_p_. Student’s *t*-test for paired measures (one-tailed when having a priori directional hypotheses) was used in the case of pairwise mean differences, using Cohen’s *d* as a measure of the effect size. Holm’s correction was applied when using multiple post hoc comparisons. Pearson’s correlation coefficients, with 95% confidence intervals (CIs), were computed to assess the degree of relationship between measures and were directional (one-tailed) when derived from a priori hypotheses. To find evidence in favor of the null hypothesis when observing non-significant differences in the main hypotheses of each study, Bayes factor (BF) was estimated using Jeffreys-Zellner-Siow (JZS) prior (Rouder et al., 2009) following the conventional interpretation of JZS values proposed by Wagenmakers et al. (2011). In addition, the recommendations by Schönbrodt et al. (2017) concerning the incorporation of prior knowledge when selecting the value of the Cauchy prior were used. Considering the observed effect sizes in our previous research (Hinojosa-Aguayo & González, 2020), which may be considered large, we decided to set the Cauchy prior to r = √2. In the case of Bayesian correlations, the value of the stretched beta prior width was set to 0.5 following the suggestions by Quintana and Williams (2018). All statistical analyses were carried out using the open-source software JASP 0.18.1 (JASP Team, 2023).

#### VMAC task

Following the exclusion criteria outlined by Garre-Frutos et al. (2024), the data from the first two trials of each block were removed, as well as trials where no response was recorded. Additionally, we filtered the data by only including responses with RTs less than 1,800 and greater than 150 (discarding less than 1% of responses in both phases). For RT analyses, we excluded incorrect responses (rewarded phase: 5.75%, unrewarded phase: 5.14%).

##### VMAC acquisition

**Fig. 1:**
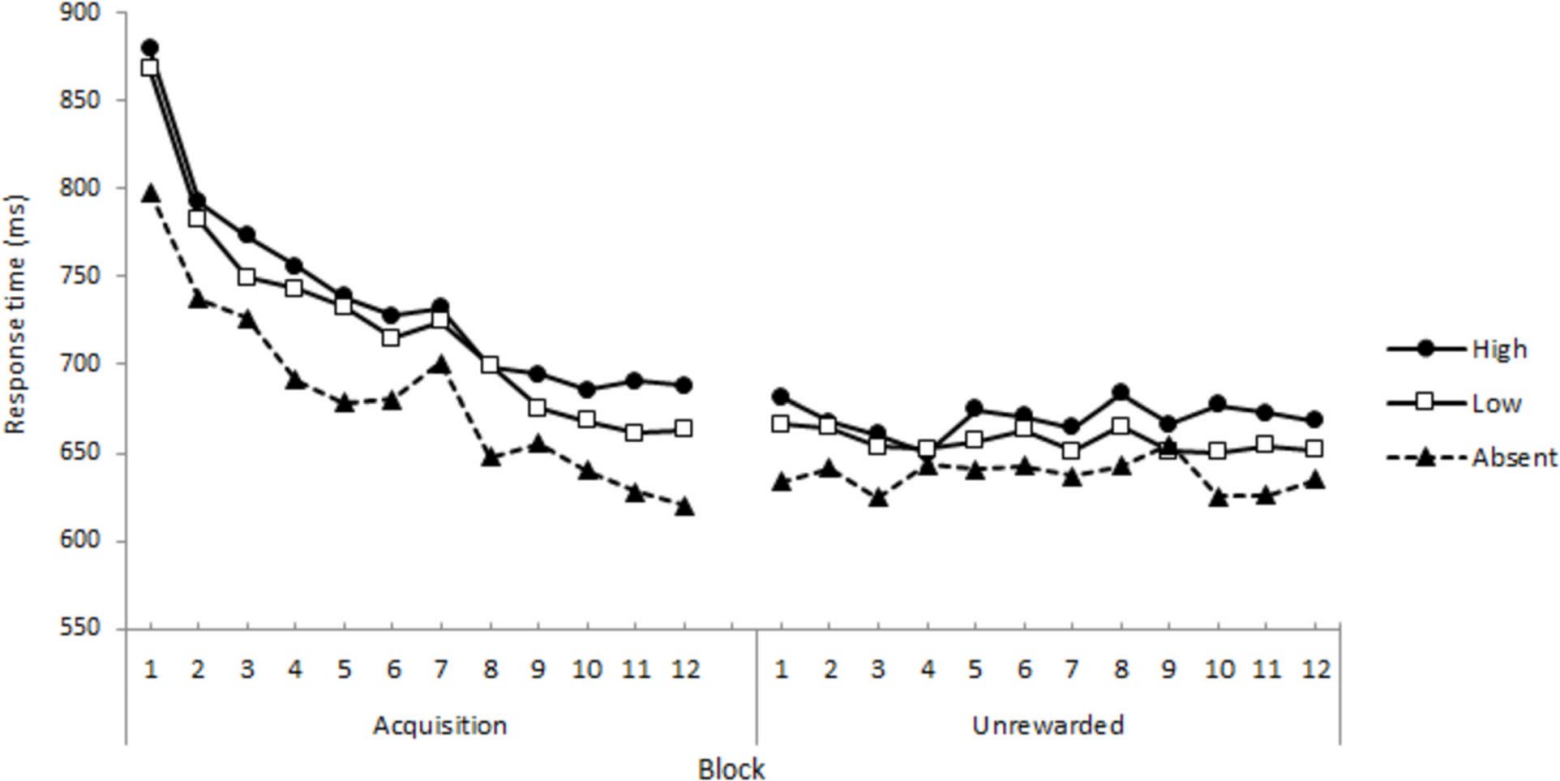
Experiment 1 VMAC task: Average correct response times by stimulus and block during the Acquisition and the Unrewarded phases

RTs for correct responses were submitted to an RM-ANOVA, with stimulus (Absent, Low, High) and block (1 to 12), as within-subject factors, yielding significant main effects of Stimulus, *F*(1.86, 106.14) = 50.83, *p* <.001, η_p_^2^ =.471, and Block, *F*(3.42, 195.04) = 37.85, *p* <.001, η_p_^2^ =.399. The interaction between these variables was not significant, *F* < 1. Post hoc comparisons using Holm’s correction revealed that the three experimental conditions differed from each other (marginal means: Absent = 683.77, Low = 723.98, High = 737.98; *SE* = 14.50), largest *p* =.011. Regarding blocks, a steady decrease in RT from the first block to the seventh (included) was observed. No further significant decreases were found from blocks 8 to 12. Therefore, the VMAC effect was evident, reaching an asymptotic value around the eight block (Fig. 1).

##### Unrewarded VMAC phase

Similarly, RTs for correct responses were submitted to an RM-ANOVA with Stimulus and Block as within-subject factors, which yielded a significant main effect of Stimulus, *F*(1.97, 112.63) = 23.65, *p* <.001, η_p_^2^ =.293 (marginal means: Absent = 637.36, Low = 656.61, High = 669.78; *SE* = 13.80). Post hoc comparisons using Holm’s corrections confirmed that the three conditions differed from each other, largest *p* =.006. No other main effect or interaction was significant, *F*_s_ < 1. This pattern of results suggests that the VMAC effect was still evident during the unrewarded phase (Fig. 1).

##### Comparison between the Acquisition and Unrewarded phases of the VMAC task

**Fig. 2:**
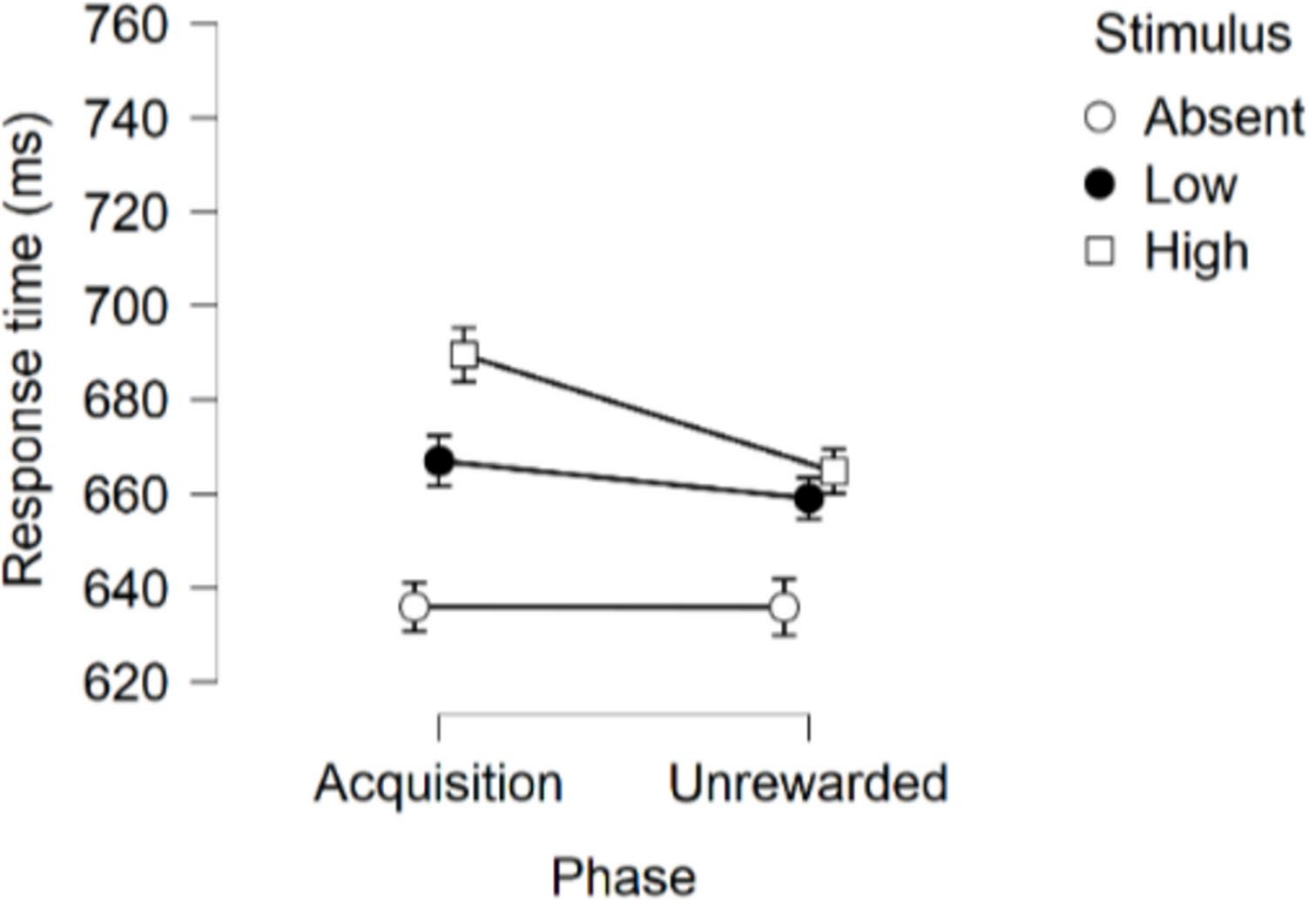
Experiment 1. Average RT by stimulus during the last four blocks of acquisition and the first four blocks of the unrewarded phase. Error bars represent SEM with the corrections applied using JASP based on Morey (2008)

As significant differences were not observed between the last four blocks of acquisition and the first four unrewarded blocks, we considered it worth comparing the RTs for each phase averaged over those four blocks (Fig. 2). The RM-ANOVA with stimulus (Absent, Low, High) and phase (acquisition, unrewarded), yielded a significant main effect of Stimulus, *F*(1.98, 113.09) = 31.44, *p* <.001, η_p_^2^ =.355, Phase, F(1, 57) = 4.03, *p* =.049, η_p_^2^ =.066, and, critically, a Stimulus × Phase interaction, *F*(1.88, 107.24) = 4.17, *p* =.020, η_p_^2^ =.068. Regarding the main effect of stimulus, the three stimuli differed from each other after applying Holm-Bonferroni correction, largest *p* =.009, with RTs being slower on High trials than the Low trials, and responses on the latter being slower than on Absent trials.

However, differences in RT were not related to accuracy in any of the phases. An RM-ANOVA conducted on the accuracy data with stimulus and block (four) as the within-subject factors revealed no significant main effects or interactions in the acquisition phase, largest *F*(2.82, 160.61) = 2.47, *p* =.07 for blocks, or in the unrewarded phase, largest *F* < 1. (Accuracy means for each stimulus during the last four blocks of acquisition: Absent = 0.95, Low = 0.95, High = 0.94; *SE* = 0.007; during the first four blocks of the unrewarded phase: Absent = 0.93, Low = 0.94, High = 0.95; *SE* = 0.008.)

To explore the critical stimulus by phase interaction in RTs, a post hoc analysis was conducted using the Holm-Bonferroni correction for multiple comparisons. We focused on the putative decrease in response time from the acquisition to the unrewarded phase for each stimulus, predicted if the attentional effect (increase in RT) produced by the High stimulus was indeed due to Pavlovian conditioning The decrease was significant for the High stimulus (mean difference of 24.76 ms, *SE* = 7.42) *p* =.007, but not for the Low stimulus, *p* = 1.000 or Absent, *p* = 1.000, for which the mean differences were 7.90 and 0.08 ms, respectively.

To compute an extinction index for the High stimulus (EXT High) to be used in relation to PIT and impulsivity measures in subsequent analyses, the difference in RT between the average of the last four blocks of acquisition (High_acq) and the first four blocks of the unrewarded phase (High_ext) was calculated for each participant. The higher the EXT High index, the more effective the unrewarded manipulation in decreasing RTs for stimulus High. As previously mentioned, we reasoned that those individuals with a lower EXT High index should show smaller outcome devaluation and specific PIT effects, as well as higher negative urgency (NU) scores. These analyses are presented at the end of this section after the analyses of the PIT task results.

#### PIT task

##### Initial level of hunger and pleasantness

The average initial level of hunger was *M* = 3.17, *SD* = 1.69. An RM-ANOVA on the pleasantness scores for the three outcomes did not yield a significant effect, *F*(1.87, 106.47) = 1.93, *p* =.153 (O1: *M* = 3.8, *SD* = 2.0; O2: *M* = 3.6, *SD* = 2.1; *M* = 3.2, *SD* = 2.1).

##### Instrumental training

As expected, there were no significant differences in the number of R1 and R2 responses performed (R1: *M* = 118.02, *SD* = 68.78; R2: *M* = 114.36, *SD* = 58.12; *t*(57) < 1, *p* =.497; BF_10_ = 0.179), or the number of O1 and O2 outcomes gained (O1: *M* = 9.38, *SD* = 2.19; O2: *M* = 9.34, *SD* = 2.33; *t*(57) < 1, *p* =.947; BF_10_ = 0.144). All participants answered the explicit R-O knowledge questions correctly before finishing this phase and proceeding to the next.

##### Pavlovian training

At the end of this phase, 51 (87.93%) participants answered all the final S-O questions correctly, five (8.62%) showed an accuracy of 75%, and two (3.45%) had an accuracy of 50%.

##### Outcome specific PIT

The average number of R1 and R2 responses made during the preCS periods was considered as the baseline rate. Total numbers of *Same* and *Diff* responses were averaged over trials and differential CS-preCS scores for each condition were compared using a one-tailed paired *t-*test that revealed significant differences between conditions, *t*(57) = 5.72, *p* <.001, *d* = 0.75 (*Same*: *M* = 5.00, *SD* = 9.26; *Diff*: *M* = −5.71, *SD* = 6.72). A one-sample *t*-test with a test value of 0 showed that both scores differed significantly from baseline (*Same*, *t*(57) = 4.18, *p* <.001, *d* = 0.54; *Diff: t*(57) = - 6.47, *p* <.001, *d* = - 0.85). This pattern of results confirms that a specific PIT effect was observed, replicating previous results with a similar task (Hinojosa-Aguayo & González, 2020, Exp. 1).

##### General transfer PIT

Differential CS-preCS scores for each condition (CS+ or S3, and CS- or S4) were computed by subtracting the average preCS number of responses from the average number of responses in the presence of each CS (collectively considering responses R1 and R2 in both cases). The difference between the differential scores for the CS+ and the CS- was significant, one-tailed, *t*(57) = 6.89, *p* <.001, *d* = 0.90 (CS+: *M* = - 0.54, *SD* = 3.82; CS -: *M* = - 6.38, *SD* = 5.94). However, only the CS- produced a change in responding compared to baseline, specifically a decrease, *t*(57) = - 8.19, *p <*.001, *d =* - 1.07. In the case of the CS + there was no significant change, *t*(57) = - 1.08, *p* =.284; BF_10_ = 0.250. This finding might be taken to indicate that S4 served as a conditioned inhibitor, decreasing the response rate when the color signaled that the vending machine was empty during the Pavlovian phase. This result again replicates previous findings when using a very similar task (Hinojosa-Aguayo & González, 2020), suggesting that S4 prompts an inhibitory PIT effect, either general or specific, without a corresponding excitatory general PIT effect for S3. While the absence of an outcome may be a very salient event in this context, participants may have understood that S3 was not linked to R1 and R2 in a significant way (they never experienced an instrumental relationship between R1 and R2 and O3) and thus they did not modify their level of responding in its presence. Therefore, we did not find evidence of a general PIT effect for S3.

##### Outcome devaluation effect on instrumental performance

The total number of non-devalued (the response whose outcome was not devalued, *Non-Dev*) and devalued (the response whose outcome was devalued, *Dev*) responses were compared using one-tailed paired-samples *t*-test that revealed a significant difference between the two measures, *t*(57) = 2.99, *p* =.002, *d* = 0.39, that is, and outcome devaluation effect (*Non-Dev*: *M* = 354.41, *SD* = 122.92; *Dev*: *M* = 261.08, *SD* = 137.73). The change in outcome pleasantness scores was also analyzed by computing the difference between the scores given at the start of the experiment and those given after the devaluation procedure. In the case of the devalued outcome, there was a significant decrease (one-tailed), *t*(57) = 5.93, *p* <.001, *d* = 0.78, (*Pre*: *M* = 3.5, *SD* = 2.1; *Post*: *M* = 2.1, *SD* = 1.8), but no change was detected in the case of the non-devalued outcome, *t*(57) = - 0.105, *p* =.542; BF_+0_ = 0.133 (*Pre*: *M* = 3.9, *SD* = 2.0; *Post*: *M* = 3.9, *SD* = 2.1). Therefore, the devaluation procedure was effective in lowering the pleasantness score of the devalued outcome.

##### Relationship between specific PIT and instrumental outcome devaluation measures

To estimate the degree of relationship between these measures, we computed three differential indices (Table 2 summarizes the correlational analyses performed in this and the following experiment). In the case of the instrumental outcome devaluation effect, we subtracted the total number of “devalued responses” from the total number of “non-devalued” responses (DEV); for the outcome-specific PIT, the average number of “Different” responses was subtracted from the average number of “Same” responses (specific PIT). As hypothesized, a significant across-task positive correlation between the specific PIT and DEV indices was found (one-tailed), *r* =.320, *p* =.007, 95% CI: [.109, 1.000]. This result replicates findings reported previously (Hinojosa-Aguayo & González, 2020, Exp. 1), suggesting that the observed specific PIT effect in this experiment is related to the instrumental outcome devaluation effect which has been proposed as a mark of goal-directed control.

**Table 2:** Summary of correlations found in Experiments 1 and 2 among measures of instrumental outcome devaluation (Dev), specific PIT, extinction of PIT (EXT PIT), VMAC (EXT High, High_acq, High_ext) and negative urgency (NU)

Experiment 1	
DEV – specific PIT	*r* =.320, *p* =.007, 95% CI: [.109, 1.000]
DEV – NU	*r* = -.333, *p* =.005, 95% CI: [-.123, −1.000]
specific PIT – NU	*n.s.*
EXT High – NU	*r* = -.233, *p* =.039, 95% CI: [-.016, −1.000]
EXT High – DEV	*n.s.*
EXT High – specific PIT	*n.s.*
Experiment 2	
EXT PIT–R-O knowledge	*n.s.*
EXT PIT– S-O knowledge	*r* =.382, *p* =.004, 95% CI: [.132,.586]
EXT PIT–Ext knowledge	*r*_*s*_ =.300, *p* =.025, 95% CI: [-.016, −1.000]
EXT PIT–Transfer knowledge	*r* =.422, *p* =.001, 95% CI: [.179,.617]
EXT PIT–NU	*r* = -.282, *p* =.017, 95% CI: [-.064, −1.000]
EXT PIT-High_acq	*n.s.*
EXT PIT- High_ext	*n.s.*
NU–High_acq	*r* =.384, *p* =.003, 95% CI: [.135,.588]
NU–High_ext	*r* =.453, *p* <.001, 95% CI: [.215,.639]

PIT = Pavlovian to instrumental transfer, VMAC = value-modulated attentional capture, DEV = instrumental outcome devaluation index (difference between the average total number of responses for the Non-Dev response and the Dev response); NU = negative urgency score; EXT High = extinction index for stimulus High (High_acq – High_ext); High_acq: average RT for correct responses across the last four blocks of the acquisition phase for stimulus High; High_ext = average RT for correct responses across the first four blocks of the extinction phase for stimulus High

##### Relationship between negative urgency, instrumental outcome devaluation and specific PIT

Also confirming our expectations, the DEV index was negatively correlated (one-tailed) with the Negative urgency (NU) score, *r* = -.333, *p* =.005, 95% CI: [- 0.123, - 1.000]. Contrary to previous findings, the correlation with positive urgency was found to be negative and significant (two-tailed), *r* = -.271, *p* =.039, 95% CI: [- 0.014, - 0.495], while no negative significant relationship between specific PIT index and NU score was found, *r* = -.032, *p* =.404, BF_+0_ = 0.137 (one-tailed).

Given that the outcome devaluation effect has traditionally served as an indicator of model-based or goal-directed processes, the specific PIT effect observed in our studies might be rooted in this type of process. When considering individual differences, it appears that the NU score plays a role in influencing this connection. Specifically, individuals with higher NU scores, indicative of emotion dysregulation, may exhibit reduced tendencies to engage in goal-directed flexible behavior. This could manifest as difficulties in effectively integrating the current incentive value of instrumental outcomes with previously acquired instrumental knowledge (i.e., R-O), that is, in implicit emotion regulation. Experiment 2 may provide further insights into this issue by examining the impact (or absence thereof) of another implicit emotion regulation process on PIT: Pavlovian extinction.

#### Relationships between specific PIT, DEV, NU, and VMAC measures

An innovative aspect of the present work was the introduction of an attentional sign-tracking measure (Albertella et al., 2020): the VMAC effect. We predicted that persistence in the VMAC effect, expressed as a lower extinction index for stimulus High (EXT High), would be related to failures in outcome devaluation and measures of impulsivity, particularly negative urgency. To test these possibilities, one-tailed Pearson correlations were estimated for the relevant variables. As hypothesized, the EXT High index was negatively correlated with NU, *r* = -.233, *p* =.039, CI: [- 0.016, −1.000]; while the correlation with the same index calculated for stimulus Low did not reach significance (two-tailed), *r* = -.044, *p* =.741, BF_10_ =.173. However, contrary to our expectations, no significant positive correlation between EXT High and either the DEV or specific PIT indices were found, highest (one-tailed), *r* =.039, *p* =.770, for DEV, BF_+0_ =.209.

This pattern of results suggests that changes in the VMAC effect due to the omission of the reward (attentional flexibility) may be impaired in participants with higher self-reported NU scores. Additionally, these findings also point to the possibility that specific PIT and VMAC effects are not necessarily associated processes – at least using our tasks. Rather, these effects may reflect different aspects of incentive salience. For instance, persistence of VMAC might be linked to earlier processing of stimulus prioritization and failure in attentional disengagement, whereas PIT could be the result of a subsequent process of response selection. Both processes appear to be related to impulsivity/compulsivity in certain individuals (Albertella et al., 2019a). Moreover, while the VMAC effect (and its persistence) may still serve as a measure of attentional sign-tracking or incentive salience in our task, specific PIT appears to represent a model-based mode of action selection, reflecting a goal-directed control process as evidenced by the observed link with the effect of outcome devaluation. Participants with better emotion regulation, and consequently lower affect-driven impulsivity, would be expected to show a more flexible process of action selection. This idea will be taken up in more detail in the General discussion.

## Experiment 2

The key differences between the designs of Experiment 2 and Experiment 1 are outlined in Table 3. In the present study, our focus was on examining the effect of extinguishing a Pavlovian cue prior to the transfer test on specific PIT by comparing this with a non-extinguished cue. Given that a failure in Pavlovian extinction is considered another form of implicit emotion dysregulation – and for this reason both experiments are thought to be complementary – our predictions were similar to those considered for the effects of instrumental outcome devaluation in Experiment 1. Therefore, we anticipated observing an effect of extinction (EXT) on specific PIT, replicating the findings reported by Seabrooke et al. (2018, Exp. 2) using our task. We expected to observe positive correlations between the effect of extinction on specific PIT and (if found again) the extinction of VMAC. Additionally, we expected to find negative correlations between NU score and indices of the extinction of both specific PIT and VMAC effects.

**Table 3:** Experiment 2: Experimental design for the outcome-specific Pavlovian to instrumental transfer (PIT) extinction task (adapted from Seabrooke et al., 2018)

Instrumental training	Pavlovian acquisition	Pavlovian extinction	Transfer test
	A – O1		
	B – O1	B – Ø	
			AD: R1 vs. R2
R1 – O1	C – O2	D – Ø	
			BC: R1 vs. R2
R2– O2	D – O2		
		E – O1	
	E – O1		
		F – O2	
	F – O2		

R = response: O = outcome; A and C = non-extinguished cues; B and D = extinguished cues; E and F = fillers

### Methods

#### Participants

Of the 76 participants who agreed to take part in the study, 19 did not complete the two tasks and were thus excluded from the analyses. Of the remaining 57 participants, we filtered out those with a response accuracy below 70% in the VMAC task. The final sample consisted of 56 participants (14 males) with an average age of 22.5 years (*SD* = 4.1, range 18–42 years), 83.93% of whom had completed university studies. The advertisement procedure and incentives for participation were the same as in the previous study.

#### Materials

The initial level of hunger and pleasantness assessment (in this case for only two outcomes), the questionnaire battery, and the VMAC task were the same as those employed in Experiment 1. Cronbach’s alpha value for the NU subscale of the UPPS-P was.85 (.68 for positive urgency). The PIT task was modified as follows.

### PIT task

In this study, we followed and adapted the design used by Seabrooke et al. (2018, Exp. 2), which appears in Table 3. Instructions provided to participants for this task can be found in the Appendix.

#### Instrumental training

The procedure was the same as that used in Experiment 1.

#### Pavlovian acquisition

Other features not mentioned in this section were identical to those in Experiment 1. In this case, six instead of four colors acted as Pavlovian cues (brown and gray were added to the previous colors) and were randomly assigned to cues A-F. Participants initially received six blocks of six trials, one for each S-O assignment, presented in a random sequence. Each block concluded with a question about one outcome-cue relationship along with feedback. Following the completion of the six blocks, participants were prompted to recall the six S-O associations, presented successively in random order without feedback. This block of questions was repeated once for all participants, and up to four times for those who failed after this point.

#### Pavlovian extinction

This took place immediately after the previous phase. The fillers (stimuli E and F) continued to be associated with outcomes O1 and O2 while stimuli B and D signaled that the vending machine was empty (no outcome). Stimuli A and C were not presented during this phase. Participants received 6 blocks of 4 trials each, presented in random order, after which they were asked about the six S-O associations, and feedback was provided for 1 s.

#### Transfer test

During this phase, conducted under nominal extinction conditions, the images of two vending machines appeared at the top and bottom of the screen in their original (uncolored) versions. Participants were informed that the position of the machines on the screen played no role in the task. However, they were instructed to pay attention to the colors of the two machines because, as they learned in the previous phase, the color provided information about the potential likelihood of a specific outcome falling when tilting the machine. When participants chose a response, both machines tilted in the same direction. This phase consisted of 24 trials with the compounds (12 for AD and 12 for BC) presented in a random order. The position of the cues (top or bottom) was counterbalanced for each compound. Other parameters, including the duration of the ITI, the baseline period (preCS in Exp. 1), and the compounds (single CSs in Exp. 1) were identical to those used in the previous study.

#### Explicit knowledge

Before finishing the experiment, participants were asked several questions in the following order.

#### S-O knowledge

*“Considering what you have learned previously in the last place, when the vending machine was lit in this color* [the image of the vending machine lit in a particular color was shown] *that meant that…”,* and three alternatives were given: “O1 (name of the snack that served as O1) was more likely to follow”; “O2 (name of the snack that served as O2) was more likely to follow”; “the machine was empty”. This was repeated for the six colors in random order.

#### Extinction

Upon the presentation of the image of the vending machine lit with a particular color, participants were asked to respond to the following question: *“During the previous phase of the experiment, did the information about the availability of the outcomes provided by this color change?”* (the response options were “Yes” and “No”). Again, this was repeated for the six colors, in random order.

#### R-O knowledge

Participants were asked the following question: “*Which key did you need to press to tilt the vending machine* [which appeared uncolored] *to obtain* *the following outcome?* [the image of one of the snacks serving as O1 or O2 was shown]. *Please press the key to answer the question.”* This question was asked for O1 and O2, in random order.

#### Transfer knowledge

The screen displayed the image of one of the two machine compounds, either AD or BC, and participants had to answer the following question: “*During the last phase of the experiment, no snacks were presented* *on the* *screen. However, when these two vending machines appeared, which snack did you expect to fall when you pressed the key …* [one of two keys, serving as either R1 or R2 was mentioned]?” The following three response options were available: “O1” [name of the snack], “O2” [name of the snack], “None”. This was repeated for each compound and response combination (four questions), in random order.

#### Strategy used during the transfer test

Finally, the participants responded to an open question intended to ascertain whether (and if so how) they used the colors of the vending machines to choose between instrumental responses taking into account their current predictive value after the extinction phase. *“There are no correct or incorrect answers* *to the next question, just try to be as honest as you can when responding. Please, could you briefly describe* *the strategy, if any, that you have followed during the last phase of the experiment?”* Participants typed their responses using the keyboard without space or time restrictions.

#### Procedure

The recruitment, materials, and procedures were identical to those used in Experiment 1 with the exceptions described above. Likewise, all phases were completed online by the participants.

### Results and discussion

#### VMAC task

As in the previous experiment, the data from the first two trials of each block were excluded, as well as trials for which no responses were recorded. We filtered the data by including responses with RTs less than 1,800 and greater than 150 (discarding less than 1% of responses in both phases). For RT analyses, incorrect responses were excluded (rewarded phase: 5.17%, unrewarded phase: 5.11%).

##### VMAC acquisition

**Fig. 3:**
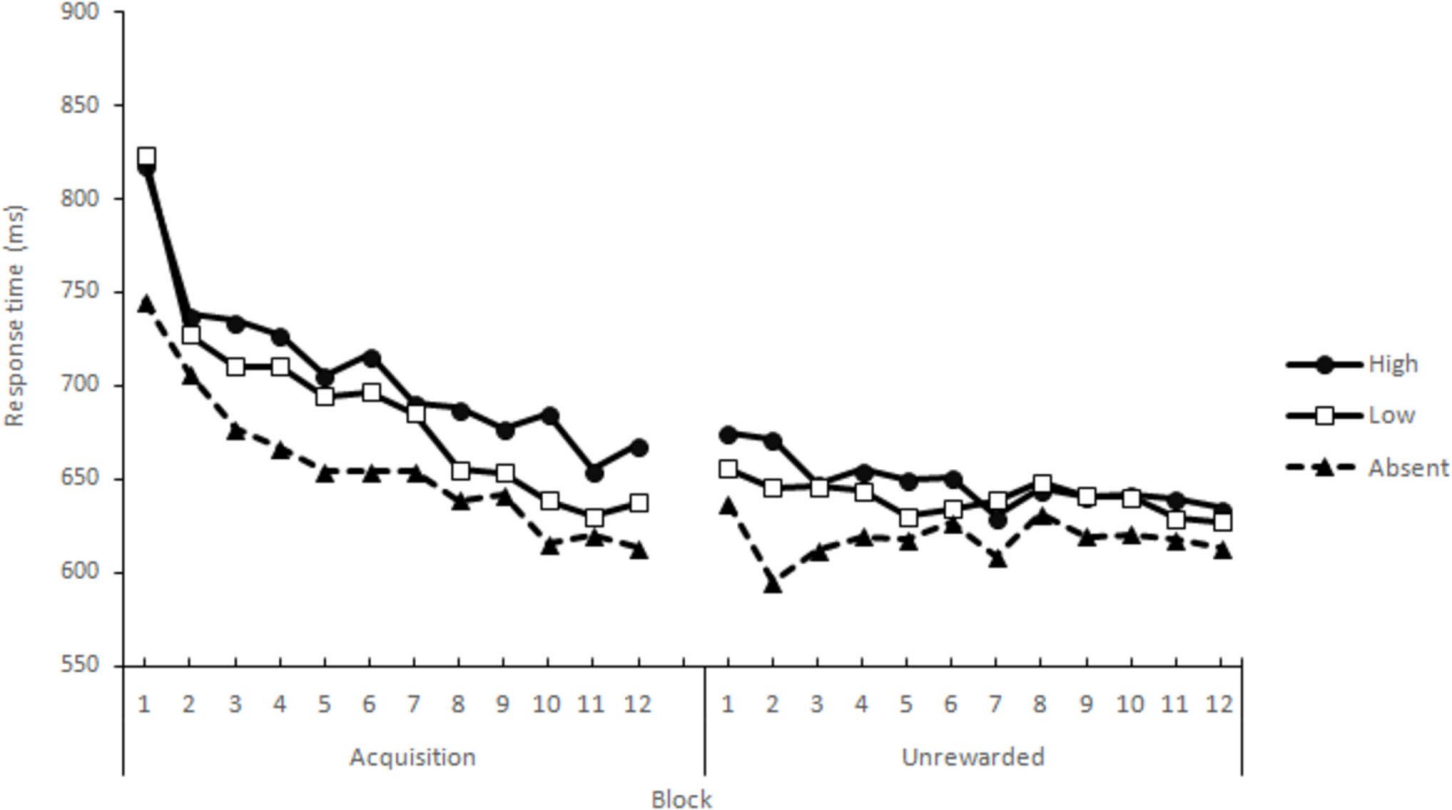
Experiment 2 VMAC task: Average correct response times by stimulus and block during the Acquisition and the Unrewarded phases

Response times for correct responses that met the inclusion criteria were submitted to an RM-ANOVA, with Stimulus (Absent, Low, High) and Block (1 to 12) as within-subject factors, yielding significant main effects of Stimulus, *F*(1.97, 108.16) = 33.90, *p* <.001, η_p_^2^ =.381, and Block, *F*(4.41, 242.96) = 22.73, *p* <.001, η_p_^2^ =.292). The interaction between these variables was not significant, *F* (8.02, 441.49) = 1.25. Regarding the main effect of Stimulus, post hoc comparisons using Holm’s correction revealed that the three conditions differed from each other (marginal means: Absent 657.28, Low 688.59, High 708.68; SE = 15.30), largest *p* =.002. Regarding blocks, as in Experiment 1, a steady decrease in RTs was observed from the first block until the seventh (inclusive), and no significant changes were found between Blocks 8 to 12 (Fig. 3).

##### Unrewarded VMAC phase

A similar analysis to that described previously yielded a significant main effect of Stimulus, *F*(1.77, 95.63) = 24.58, *p* <.001, η_p_^2^ =.313 (marginal means: Absent = 617.07, Low = 638.21, High = 647.69; SE = 13.35). Post hoc comparisons using Holm’s corrections revealed that the three conditions differed from each other, largest *p* =.036. No other main effect or interaction was significant, largest *F* (8.04, 434.33) = 1.32 *p* =.231 (Fig. 3).

##### Comparison between Acquisition and Unrewarded phases

As in the previous experiment, since no significant differences were detected between the last four blocks of acquisition or the first four blocks of the unrewarded phase, RTs were averaged across blocks, and the means submitted to an RM-ANOVA with Stimulus (Absent, Low, High) and Phase (Acquisition, Extinction) as within-subject factors. This analysis revealed a significant main effect of Stimulus, *F*(1.97, 108.177) = 40.64, *p* <.001, η_p_^2^ =.425 (marginal means, Absent = 619.35, Low = 643.88, High = 666.83; SE = 13.82); while the main effect of Phase was not significant, *F* < 1. The Stimulus × Phase interaction, *F*(1.51, 82.84) = 1.68, *p* =.198, did not reach significance, indicating that omission of the reward did not modify the VMAC effect.

Finally, as in the previous experiments, the differences found in RTs did not correspond to differences in accuracy. Two separate RM-ANOVAs conducted on the data from last four blocks of acquisition and the first four blocks of the unrewarded phase revealed no main effects or interactions; acquisition phase, largest *F*(1.83, 100.48) = 2.20, *p* =.120 (Absent 0.96, Low 0.95, High 0.95, SE = 0.007), unrewarded phase, *F*(1.58, 86.89) = 1.24, *p* =.289 (Absent 0.93, Low, 0.95, High 0.95; SE = 0.008).

#### PIT task

##### Initial level of hunger and pleasantness

The average initial level of hunger was *M* = 3.2, *SD* = 1.8. A paired *t*-test conducted on the pleasantness scores for the two outcomes serving as O1 and O2 revealed no significant differences between them, *t*(55) = 1.41, *p* =.164, BF_10_ = 0.372 (O1: *M* = 4.1, *SD* = 2.1; O2: *M* = 3.7, *SD* = 1.9).

##### Instrumental training

There were no significant differences between the number of responses performed as R1 or R2 (R1: *M* = 107.30, *SD* = 52.92; R2: *M* = 110.64, *SD* = 45.34; *t*(55) < 1, *p* =.624; BF_10_ = 0.164) or the number of outcomes gained, O1 or O2 (O1: *M* = 8.96, *SD* = 2.36; O2: *M* = 9.30, *SD* = 2.44; *t*(55) < 1, *p* =.593; BF_10_ = 0.167). All participants answered the explicit R-O knowledge correctly before finishing this phase, as this was a requirement for proceeding to the next phase.

##### Pavlovian acquisition

The percentage accuracy on the S-O questions during this phase was 93.15% after the first block of questions and 94.64% after the second. Six participants needed a third block, and four of them needed a final fourth block.

##### Pavlovian extinction

Regarding the final explicit questions after the Pavlovian phase, the average percentage accuracy was 93.60%.

##### Transfer test

**Fig. 4:**
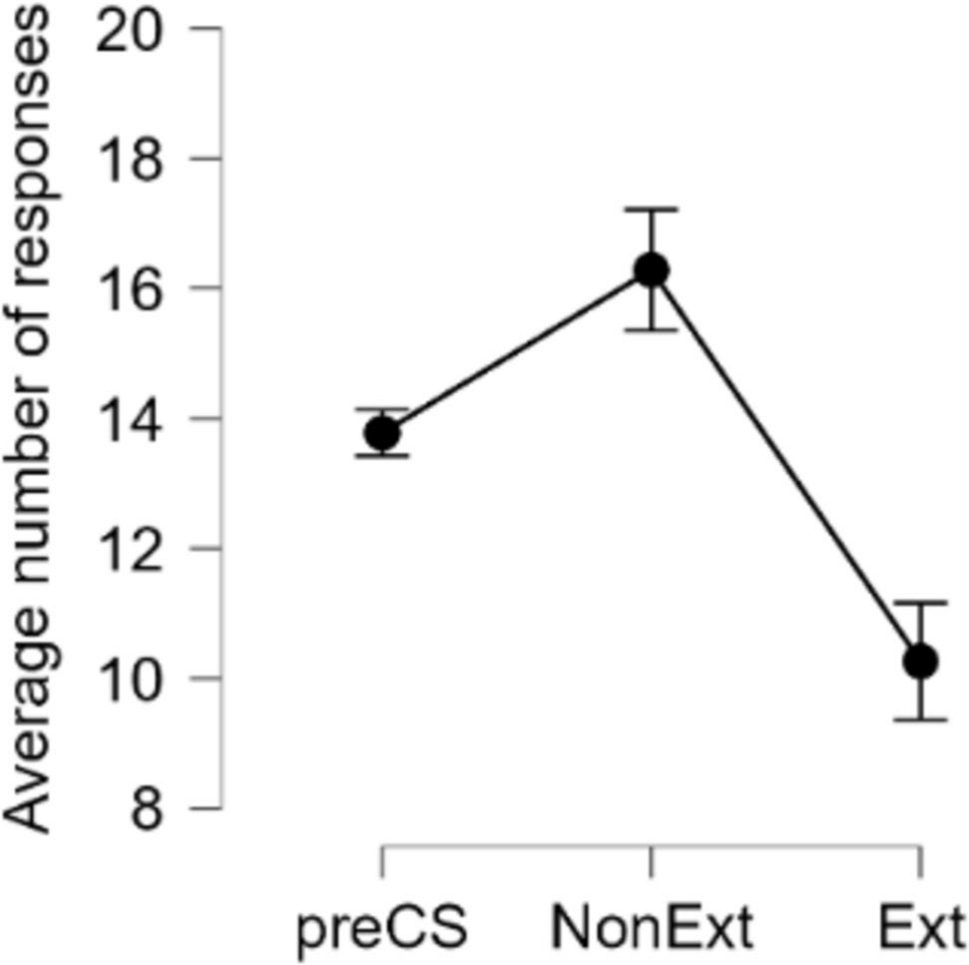
Experiment 2. PIT test for the AD and BC compounds after Pavlovian extinction. Average number of responses performed during the three 6-s periods: pre-compound (preCSs), Non-Extinguished CS (**NonExt: A & C**), and Extinguished (**Ext B & D**). Error bars represent SEM with the corrections applied using JASP based on Morey (2008)

For each compound, AD and BC, the average number of R1 and R2 responses for each CS was recorded. For each compound, the response whose outcome-related cue had not undergone extinction was then labeled “Non-extinguished (NonExt),” whilst the alternative response was labeled “Extinguished (Ext).” The average number of responses for both categories was then calculated across the compounds (Fig. 4). A paired *t*-test (one-tailed) showed that the number of NonExt responses was higher than that of Ext responses, *t*(55) = 4.11, *p* <.001, *d* = 0.55 (NonExt: *M* = 16.28, *SD* = 6.99; Ext: *M* = 10.26, *SD* = 6.06). Moreover, the average number of NonExt responses was significantly higher than the average number of responses performed during the pre-compound periods (two-tailed) *t*(55) = 2.83, *p* =.006, *d* = 0.38, while the average number of Ext responses was significantly lower, *t*(55) = - 4.29, *p* <.001, *d* = - 0.57 (pre-compound number of responses: *M* = 13.78, *SD* = 2.74). These results confirm that specific PIT was affected by the extinction of Pavlovian cues. Along with the effect of instrumental outcome devaluation observed in Experiment 1, these results are compatible with the idea that the mechanism underlying the specific PIT effect exhibited by participants in our studies may be controlled by a propositional, goal-directed process (see, e.g., Seabrooke et al. 2018).

##### Explicit knowledge after the transfer test

There was some variability among participants with respect to the level of explicit knowledge of the different assessments reported at the end of the experiment. The average level of accuracy regarding knowledge of the final S-O associations (current Pavlovian knowledge including information acquired during the Pavlovian extinction phase) was 85.42%; the average accuracy when detecting that the predictive value of a particular color, or Pavlovian cue, had changed (or not) was 88.39 % (extinction knowledge); while knowledge of the R-O assignation during the instrumental phase (instrumental knowledge) was slightly lower at 79.46%. Finally, average accuracy related to the question about the response to be performed during the transfer test taking into account the current predictive value of the Pavlovian cue and the corresponding response with which it shared the outcome (transfer knowledge) was the lowest at 53.12 %. This latter figure suggests that, on average, most participants may have been choosing between R1 and R2 randomly, and that this test appeared to be extremely difficult. However, according to the open question about the strategy used, 21 out of 56 participants accurately reported having chosen the response for which the delivery of the outcome was signaled by the non-extinguished cue, producing a general extinction effect when comparing the overall means.

##### Relationship between explicit knowledge and transfer test performance

An index of performance on the transfer test taking into account the effect of extinction of the Pavlovian cues (EXT) was calculated by subtracting the average number of Ext responses from that of the NonExt responses for each participant. The higher the index, the larger the effect of extinction on specific PIT and therefore, the better the performance on the test. We reasoned that the level of performance on the transfer test may depend on the accuracy of explicit knowledge exhibited by participants, particularly regarding the predictive S-O and R-O relationships, and the integration of this information at the time of decision making during the transfer test. Confirming these expectations, the EXT index was significantly and positively correlated with the measures taken at the end of the transfer test on S-O knowledge, *r* =.382, *p* =.004, 95% CI: [0.132, 0.586], extinction knowledge (learning that during the experiment a particular Pavlovian cue had changed its predictive value or not), Spearman’s *rho* =.300, *p* =.025, 95% CI: [0.040, 0.522], and the transfer knowledge (to determine which response was related to the outcome that was still present, as signaled by the non-extinguished cue), *r* =.422, *p* =.001, 95% CI: [0.179, 0.617]. However, performance on the transfer test appeared to be unrelated to R-O instrumental knowledge, *r* =.234, *p* =.082, 95% CI: [- 0.031, 0.468]. This might be unsurprising, given that participants received extra training if they failed the R-O questions, thus reducing variability among participants.

##### Relationship between the effect of extinction of the Pavlovian cues on PIT and measures of impulsiveness

**Fig. 5:**
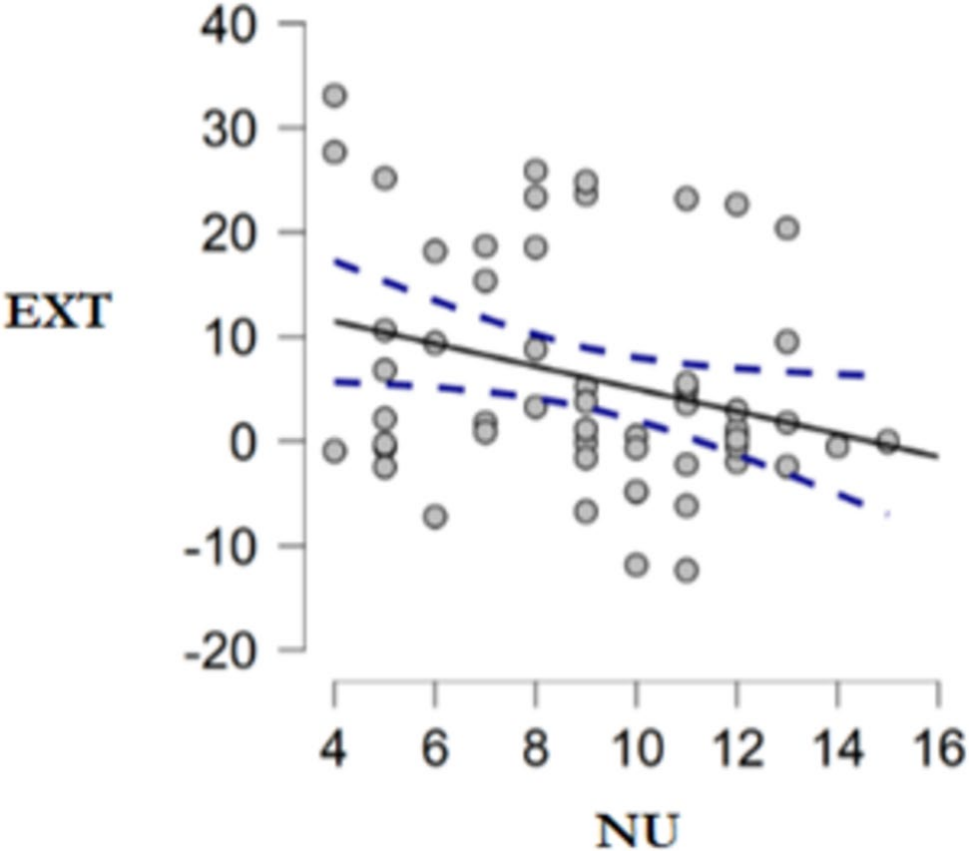
Experiment 2. Pearson correlation scatter plot between EXT index (PIT task) and NU score. Linear regression (continuous line) with its confidence interval (dotted lines)

The EXT index was significantly and negatively correlated with negative urgency (NU), one-tailed *r* = -.282, *p* =.017, 95% CI: [−1.000, - 0.064], as predicted (Fig. 5). An exploratory analysis found no significant relationship with positive urgency, two-tailed *r* = -.219, *p* =.105, BF_10_ = 0.602.

##### Relationship between VMAC effects and both specific PIT and negative urgency

Despite the failure to observe extinction of the VMAC effect when removing the reward in the present experiment, we reasoned that those participants with higher RTs for stimulus High during the final part of the acquisition and/or the first blocks of the unrewarded phases could be considered to exhibit more attentional sign-tracking. Therefore, we used High_acq and High_ext indices^4^ and calculated correlations with the key elements of the specific PIT effect and NU score. Since this was not an a priori prediction, the analyses were two-tailed. Both indices (High_acq and High_ext) showed significant positive correlations with NU *r* =.384, *p* =.003, CI: [0.135, 0.588], and *r* =.453, *p* <.001, CI: [0.215, 0.639], respectively. No significant correlations were observed between VMAC indices and the index of the effect of Pavlovian extinction (EXT) on specific PIT, largest *r* = -.112, *p* =.411, BF_10_ = 0.232.

This pattern of results adds evidence to the proposal that emotion dysregulation as a trait is positively related to attentional sign-tracking and its persistence.

## General discussion

One of the primary goals of this research was to explore the relationship between emotion dysregulation – specifically negative urgency as a trait and failures in implicit emotion regulation processes (outcome devaluation and extinction) – and the extent to which individuals attribute incentive salience to reward cues. Incentive salience was assessed using tasks designed to evaluate (1) value-modulated attentional sign-tracking (VMAC) and (2) reward-cue influence on action control and selection (PIT). In both cases, indicators of difficulties in updating behavior were measured by the persistence of behavior following changes in outcome value (Experiment 1) and cue-outcome contingency (Experiment 2) in the PIT task, as well as extinction in both experiments in the VMAC task. To our knowledge, no previous study has examined the relationship between these various measures in relation to emotion dysregulation. Below, we discuss findings related to several specific objectives aimed at achieving this overarching goal.

### Attentional sign-tracking

The VMAC effect, a proxy for attentional sign-tracking, was consistently observed during the acquisition phase in both of our experiments, confirming that a cue for reward captured attention even when participants were explicitly instructed to ignore it and the cue itself had no instrumental role in the task. The analysis of the impact of reward omission during the second phase yielded a mixed pattern of results.

First, considering the entire unrewarded phase in both experiments, the VMAC effect was sustained throughout the blocks, suggesting that the effect persisted even when the cue was no longer predictive of an increase in reward (or even a reward at all). Our pattern of results adds to previous evidence indicating the persistence of the effect even when the prospect of reward is eliminated (Garre-Frutos et al., 2024; Watson et al., 2019), signaling that VMAC is an automatic process rather than the result of an explicit strategy directed toward an informative stimulus. This is consistent with the VMAC task being viewed as a tool for estimating individual differences in the propensity to exhibit the attentional sign-tracking pattern, which has been linked to a susceptibility to impulse control issues such as addictive-like behaviors, compulsive behaviors, impulsivity, and risky decision-making (Anderson, 2021; Colaizzi et al., 2020).

However, closer inspection of the data comparing equal-length intervals of both phases (average final four blocks of the rewarded and average first four blocks of the unrewarded phase) showed a decrease in RT in the case of stimulus High (but not for the others) in Experiment 1, indicative of a potential reduction of attentional sign-tracking between phases. Moreover, the extinction index was negatively correlated with negative urgency, suggesting that the persistence of VMAC after the omission of the reward might be more pronounced in participants with higher NU scores. Although this pattern was not found in Experiment 2, the two average four-block RTs calculated for stimulus High at the end (High_acq) and start (High_ext) of each phase, while not statistically different this time, maintained significant positive correlations with NU. This again points to subtle individual differences in attentional sign-tracking and its persistence depending on this emotion dysregulation trait. Participants with higher NU scores showed longer RTs in the presence of the High stimulus in significant intervals of the two phases. We acknowledge that our results provide only limited and rather arbitrary evidence (the study was not preregistered, and these measures were selected using a somewhat exploratory search).

Nonetheless, considering that, as in the studies of Watson et al. (2019) and Garre-Frutos et al. (2024), our participants were not specifically selected based on their NU scores or the presence of a given behavioral disorder, our findings point to the relevance of measuring individual differences in emotion dysregulation in so-called healthy participants, allowing preclinical approaches to the issue. More research is needed to pursue this potential connection between attentional sign-tracking and affect-driven impulsivity in the general population.

The adverse impact of emotion dysregulation on cue reactivity and action control and selection is expected to be stronger in clinical or subclinical samples. Failures in the extinction of a cue-reward association may lead to generalized incentive salience of cues when salience should be attenuated. For instance, participants with different levels of craving and gambling severity show impaired extinction, but not acquisition, of conditioned responses to erotic and gambling-related cues depending on their NU scores, which also predicted higher craving scores (Quintero et al., 2020; for related results involving positive urgency, see Muela et al., 2023).

It is important to note that investigation in the general population, as well as those including participants with subclinical or clinical disorders, may benefit from increasing the reliability of the VMAC measure, as measures with poor reliability are not sensitive in detecting individual differences. This improvement is needed to establish a link between individual differences in emotion dysregulation and behavioral disorders (for specific suggestions, see Garre-Frutos et al., 2024). Alternatively, the use of other measures showing superior reliability, such as those based on eye tracking, and the increase of sample sizes and the number of trials in behavioral tasks, are warranted to maximize statistical power, reliability, and reproducibility (Kim et al. 2025). Increasing reliability (and thus validity) will favor the use of experimental tasks beyond those relying on self-reported measures of emotion dysregulation based on declarative knowledge. For instance, future studies could aim to provide further evidence of the validity of VMAC as a measure of attentional sign-tracking, for example by examining its association with other theoretically related measures. Note that although there are several studies showing that VMAC is related to self-report measures of impulsivity, depression symptoms, and other psychopathological conditions (see Anderson, 2021), it is sometimes difficult to find associations between experimental tasks that purport to measure the same construct (Hedge et al., 2018; Nebe et al., 2024). Finding more evidence for validity will allow us to explore the role of incidental emotion regulation processes whose outcomes may lie outside of conscious recollection.

### Reward-seeking behavior (PIT)

No significant relationships were observed between VMAC and PIT measures in either experiment. This pattern of results suggests that, at least with the tasks and measures employed in our study, specific PIT and attentional sign-tracking – considered measures of incentive salience – are not necessarily related, being differentially affected by emotion dysregulation, promoting attentional sign-tracking and impairing specific PIT.

The key results regarding specific PIT include its significant positive correlation with the instrumental outcome devaluation effect (Exp. 1) and the success in finding an effect of Pavlovian extinction (Exp. 2) on specific PIT. These results replicate those reported previously (Hinojosa-Aguayo & González, 2020, Exp. 1; Seabrooke et al., 2018, Exp. 2), and suggest that specific PIT may potentially be a goal-directed process, sensitive to changes in outcome-value and cue-outcome contingency. Both instrumental outcome devaluation and Pavlovian extinction have been proposed as instances of implicit emotion regulation processes (Etkin et al., 2015), which act in a rather automatic way before the emotion itself is fully appraised. The observation that both effects were significant and negatively correlated with NU is consistent with this framework and further suggests that the extent to which specific PIT may rely on goal-directed control depends on individual differences in emotion dysregulation, particularly affect-driven impulsivity, even in a healthy sample. Thus, specific PIT might be more or less automatic depending on the success of incidental emotion regulation.

This possibility may fit well with dual-process models of associative learning in which, for instance, the PIT learning phenomenon may reflect the formation of an automatic associative S-O-R link mechanism, as well as higher order propositional reasoning processes. The automatic process will be dominant under circumstances when reasoning is less likely, such as when using complex designs in which the number of Pavlovian and instrumental contingencies is high or there are reversal instructions and multiple outcomes associated with each response (e.g., de Wit et al., 2013; Seabrooke et al., 2019). Likewise, emotion dysregulation would be expected to deplete cognitive resources and foster automatic rather than goal-directed control processes. A more extreme consideration is encouraged by pure propositional theories of human PIT. According to these theories, specific PIT involves controlled goal-directed actions based on the explicit knowledge of Pavlovian and instrumental contingencies (for a detailed account, see, e.g., Mahlberg et al., 2021). Specific PIT is thought to be the consequence of a controlled decision-making process whereby participants infer which outcome is more available, as signaled by the cues, and thus which instrumental response is more likely to be reinforced on a given trial (Seabrooke et al., 2018). Evaluation of the theoretical accounts of the PIT effect falls beyond the scope of the present work. However, some of our results are consistent with the idea that the effect in humans reflects the operation of goal-directed control, which is related to explicit knowledge of contingencies involving the outcome, at least in participants with lower emotion dysregulation scores. Similarly, using essentially the same task, we previously found an effect of outcome devaluation on specific PIT that depended on the negative urgency score (Hinojosa-Aguayo & González, 2020, Exp. 2).

Our findings indicating that a propensity for poorly regulated negative emotions may impair outcome devaluation, that is, goal-directed control, align with prior research demonstrating the impact of negative emotions on reward-seeking behavior. Notably, studies on acute stress induction in humans have revealed detrimental effects on goal-directed control of instrumental choice, leading to impaired instrumental outcome devaluation and a shift towards habitual behavior (e.g., Schwabe & Wolf, 2009). Similarly, negative emotional appraisal appears to selectively hinder the retrieval of an expected instrumental outcome value from working memory, a phenomenon observed even when accounting for factors such as task disengagement, ineffective devaluation treatment, or poor explicit knowledge of the response-outcome association (Pritchard et al., 2018).

Emotion regulation processes help us to focus on long-term interests over immediate emotional needs, and their failures therefore impact the control of action selection. Indeed, participants with high NU scores have been reported to prefer immediate solutions when experiencing negative emotions due to the cognitive cost of dealing with impulse control or the difficulty of using effortful and long-term emotion regulation. Instead, they employ non-adaptive emotion regulation strategies in the pursuit of short-term emotional relief or avoidance of negative emotional states. Reliance on these types of strategies can be damaging in the long term, resulting in both internalizing and externalizing disorders (King et al., 2018).

### Limitations

As with other studies focusing on individual differences, we mainly rely on correlational methodology, and the results should be interpreted with this in mind. Our studies were not formally preregistered; however, their main goals were theoretically driven. Several of these were attempts to replicate findings from our previous research (Hinojosa-Aguayo & González, 2020) and from other laboratories (Morris et al., 2015; Quail et al., 2017; Seabrooke et al., 2018; Watson et al. 2019). These replications included the detection of the behavioral effects of specific PIT, instrumental outcome devaluation, and the effects of Pavlovian extinction on the specific PIT and VMAC effects, thus contributing to the generalizability of those results. Although we did not conduct an a priori sample-size estimation, we endeavored to increase sample sizes compared to those used in previous published studies whenever possible.

The studies were conducted online for convenience following the COVID-19 pandemic (for other related experimental studies conducted online, see also Garre-Frutos et al., 2024; Le Pelley et al., 2022; Watson et al., 2020). Overall, we observed results very similar to those of studies conducted in laboratory settings in the case of PIT and instrumental outcome devaluation effects. Concerning the VMAC task, we generally found results identical to those of previous studies during the acquisition phase but observed more varied results in the unrewarded phase. One notable advantage of using online recruitment was the increased heterogeneity of our samples, which included members of the university community beyond students of Psychology, thereby enhancing generalizability.

Participants consistently completed the VMAC task before the PIT task. Therefore, we did not control for any potential order effect. This choice was guided by the rationale that the VMAC task was longer and more attentionally demanding than the PIT task. However, it is unlikely that the first task had a considerable effect on the second, as they were conducted at least 24 h apart.

We primarily followed extinction procedures used in previous research in which no points were available during the unrewarded phase. This method may be rather insensitive, and the persistence observed in VMAC could result from the task’s inability to effectively engage the extinction process. Additionally, feedback on performance might have sustained behavior during the unrewarded phase. Future investigations should consider these points. For instance, employing more sensitive extinction procedures (e.g., eliminating the extra points for stimulus High during the second phase) or using more salient manipulations like reversal learning (Albertella et al., 2019b), where persistence despite reversal has been linked to risky patterns of alcohol use.

## Conclusions

Taken together, our results highlight the impact of individual differences in emotion dysregulation, specifically negative urgency (an affect-driven impulsivity factor), on processes involving the attribution of incentive salience to reward cues, as well as on action control and selection. Regarding incentive salience measured as attentional sign-tracking, individuals with higher levels of negative urgency also showed higher RTs for the stimulus signaling an increase in reward and demonstrated greater persistence of the VMAC effect during the unrewarded phase. This occurred even if participants were explicitly instructed to disregard the stimulus during acquisition and when the prospect of reward was eliminated during the second phase of training. This pattern of results suggests that attentional sign-tracking may be an automatic process rather than the product of an explicit strategy of controlled attention toward the stimulus as an informative cue. Negative urgency was also related to lower levels of instrumental outcome devaluation and Pavlovian extinction (both considered forms of implicit emotion regulation) in the PIT task. Moreover, no relationships were found between specific PIT (or its extinction) and attentional sign-tracking, suggesting that these phenomena are governed by different mechanisms. Unlike VMAC, the PIT effects observed in our experiments appear to be goal-directed and sensitive to changes in outcome value and cue-outcome contingency, at least in individuals with lower affect-driven impulsivity. In the case of Pavlovian extinction, the effect was linked to the extent of explicit knowledge involving the relevant contingencies, further emphasizing the controlled nature of this phenomenon in our task.

## Supplementary Information

Below is the link to the electronic supplementary material.

Supplementary file1 (DOCX 24 KB)

## Footnotes

1 For the sake of brevity, we will use the term “VMAC task” to refer to the visual search task designed to estimate the VMAC effect.

2 Please note that the outcome devaluation phase occurred after the PIT phase. Therefore, the devaluation effect we intended to estimate here was on instrumental performance, not on PIT.

3 As part of a wider study, participants were presented with two other questionnaires. However, only the urgency subscales of the short Spanish version of the UPPS-P were relevant to the present studies and the data for the rest are omitted. The questionnaires were the Spanish adaptation of the Emotion Regulation Questionnaire (ERQ); and the Spanish Version of the Shortened Sensitivity to Punishment and Sensitivity to Reward Questionnaire (SPSRQ-20).

4 Please note that High_acq and High_ext RTs refer to the average RT of the last four blocks of the acquisition phase and the first four blocks of the unrewarded phase, respectively. These were the components of the EXT High index in Experiment 1.
