# Supplementary material for: Extinction of outcome-specific Pavlovian-to-instrumental transfer (PIT), instrumental outcome devaluation, and reward-related attentional capture are predicted by affect-driven impulsivity
Source: Learn Behav. 2025 May 14;53(4):335–54. doi: 10.3758/s13420-025-00676-1 (PMC12615517; doi:10.3758/s13420-025-00676-1)
Supplement: Supplementary file 1 — Supplementary file1 (DOCX 24 KB) [file 13420_2025_676_MOESM1_ESM.docx]

**Appendix**

**1. VMAC task instructions translated to English**

Welcome to the experiment.

Before starting, it is necessary that you concentrate and avoid any kind of distraction since it is important to control the noise that may be produced by the context in which each person performs the task. Also, remember that depending on your performance you will be able to earn real money. Any kind of distraction will affect you negatively. Therefore, we ask you to perform the experiment in a place with a good internet connection, as isolated as possible, and dim lighting. In addition, please turn off, block, or keep your cell phone out of reach during the experiment.

Press S if you have followed the instructions.

[Instructions to calibrate participant’s distance from the screen using the virtual chinrest procedure devised by Li et al. (2020)]

**VMAC. Acquisition**

You are about to perform a visual search task. In this task we will introduce you to a configuration like this one throughout the experiment:

[configuration is shown]

Press the space bar to continue.

You will encounter different stimuli with two possible shapes: Diamonds or Circles. Your task is to respond to the orientation of the line within the diamond while trying to ignore the other shapes. In turn, the line within the diamonds may have two orientations: Vertical or Horizontal. If the orientation of the line inside the diamond is vertical press H. If the orientation of the line inside the diamond is horizontal press V.

Before starting the experiment, we are going to perform a few practice trials to familiarize yourself with the task. Remember:

Press H if the orientation of the line inside the diamond is vertical.

Press V if the orientation of the line inside the diamond is horizontal.

In case you want to read the instructions again, press the R key. On the other hand, if you want to start practicing, press the space bar.

[practice trials completed]

You have completed the practice phase Very good! Now that you are familiar with the task, let's get started with the actual experiment.

Press the space bar to continue.

From now on, throughout the task you will be able to earn different amounts of points depending on your performance. Answering correctly will lead you to earn points, but mistakes will cause you to lose the same number of points you would have earned. In addition, answering faster can make you earn (if you get it right) or lose (if you miss) more points. Also, if you answer too slowly, you will not earn points.

Press the space bar to continue.

Finally, do you remember that sometimes one of the circles could appear in another color? From now on, the color of the circle will determine the number of points you earn.

If a circle appears in color [Color High], you can earn 10 times more points in that trial. On the other hand, if it appears in color [Color Low], you will not earn extra points:

Press the space bar to continue.

Now you are about to start the experiment.

Remember:

Press H if the orientation of the line inside the diamond is vertical.

Press V if the orientation of the line inside the diamond is horizontal.

In case you want to read the instructions again, press the R key, if you want to start the experiment, press the space bar.

**VMAC. Extinction**

You have done very well!

You will continue with the task but, from now on, you will no longer earn points. Therefore, the color in which some stimuli may appear is completely irrelevant.

If you have read and understood this, press S to continue with the experiment.

**2. Pavlovian-to-instrumental task: Instructions**

**Experiment 1. Instructions translated to English.**

Instrumental training:

You are part of a group trying to obtain products to celebrate birthdays for underprivileged children. Any food that can be obtained will be greatly useful. There's a rumor that free snacks can be obtained from a vending machine. Press the ‘up arrow’ and ‘down arrow’ keys of the keyboard to move the machine left and right. Use only the index finger of your dominant hand. Shake the machine until a product falls out. You have to learn which snack falls when you use the’ up arrow’ and which when you use the ‘down arrow’ keys. Occasionally, a question will appear to test your knowledge about this relationship.

Pavlovian training:

Your group has discovered that the machines light up and provide information, indicating if the machine is too full of a product, making it fall more easily, or if the machine is empty. You must solely observe and pay attention to learn how each color relates to each possible outcome. Again, occasionally, what you have learned will be assessed through a question. Use the keyboard [numbers 1, 2, 3, or 4] to give the correct answer.

[1, 2, and 3 means that the machine is plenty of either O1, O2, or O3; 4 means that the machine is empty at that moment]

Transfer test:

Now your group and you are going to show the knowledge you have acquired. The purpose of this phase is to optimize the procedure of acquisition of snacks before you go out on the streets.

Remember that you can obtain food by shaking the machine from side to side using the ‘up arrow’ and ‘down arrow’ keys, just as you learned in the first phase. Again, use only the index finger of your dominant hand. Depending on which key you press, you will get one product or another. However, in this phase, the images of the snacks will not appear on the screen, although your task remains to gather as much food as you can, as efficiently as possible.

Furthermore, keep in mind that the machines will occasionally light up with different colors. These colors will indicate which products are more likely to fall at that particular moment, as you learned in the second phase.

In conclusion, press the "up arrow" and "down arrow" keys throughout the task to obtain food, as you did in the first phase. Similarly, take into account the colors that appear occasionally to know which foods would be more likely to fall, according to what you have learned in the second phase. Try to gather as much food as you can so that underprivileged children can celebrate the best birthdays of their lives.

Press the spacebar to continue.

Outcome devaluation phase:

Congratulations, you've passed the test and you're out on the streets with your group trying to get products for underprivileged children. You're in an area with quite a few vending machines, so it seems like a good spot. However, one of you has discovered that the machines are infected! Disgusting bugs have invaded some of the packages of one of the food items. When you shake the machine, one of the products that falls out will have to be shared with these new tenants. It's not all the packages of that food, but half of them. Below, you can see a sample of the condition half of the packages of that infected product are in. Take a good look at the image for the amount of time provided.

[the gif image of the outcome and cockroaches is shown]

This is a problem because, on one hand, you need to get as much food as you can, and on the other hand, half of the packages of the food item shown to you are infected with insects. Remember that you can get food by shaking the machine to the left or to the right using the ‘up arrow’ and ‘down arrow’, just as you learned in the first phase. Once again, use only the index finger of your dominant hand.

Depending on which key you press, you'll get one product or another. However, in this phase, images won't appear on the screen, although your task remains to gather as much food as you can, as efficiently as possible. Go ahead, hit the space bar to continue and get products for the underprivileged children.

**Experiment 2. Instructions translated to English.**

Instrumental training:

You are part of a group trying to obtain products to celebrate birthdays for underprivileged children. Any food that can be obtained will be greatly useful. There's a rumor that free snacks can be obtained from a vending machine. Press the ‘up arrow’ and ‘down arrow’ keys of the keyboard to move the machine left and right. Use only the index finger of your dominant hand. Shake the machine until a product falls out. You have to learn which snack falls when you use the’ up arrow’ and which when you use the ‘down arrow’ keys. Occasionally, a question will appear to test your knowledge about this relationship.

Press the spacebar to continue.

Pavlovian training:

Your group has discovered that the machines light up and provide information, indicating if the machine is too full of a product, making it fall more easily, or if the machine is empty. You must solely observe and pay attention to learn how each color relates to each possible outcome. Again, occasionally, what you have learned will be assessed through a question. Use the keyboard [numbers 1, 2, or 3] to give the correct answer.

[1 and 2 means that the machine is plenty of either O1 or O2; 3 means that the machine is empty at that moment]

Transfer test:

Now your group and you are going to show the knowledge you have acquired. The purpose of this phase is to optimize the acquisition of snacks, and for this, we have gathered two machines, which will allow you to gain more products.

Remember that you can obtain food by shaking the machine from side to side using the ‘up arrow’ and ‘down arrow’ keys, just as you learned in the first phase. Again, use only the index finger of your dominant hand. Depending on which key you press, you will get one product or another. However, in this phase, the images of the snacks will not appear on the screen, although your task remains to gather as much food as you can, as efficiently as possible.

Furthermore, keep in mind that the machines will occasionally light up with different colors. These colors will indicate which products are more likely to fall at that particular moment, as you learned in the second phase. The location of each machine—top or bottom—does not provide any relevant information.

In conclusion, press the "up arrow" and "down arrow" keys throughout the task to obtain food, as you did in the first phase. Similarly, take into account the colors that appear occasionally to know which foods would be more likely to fall, according to what you have learned in the second phase. Try to gather as much food as you can so that underprivileged children can celebrate the best birthdays of their lives.

Press the spacebar to continue.
